# A Virally Encoded DeSUMOylase Activity Is Required for Cytomegalovirus Reactivation from Latency

**DOI:** 10.1016/j.celrep.2018.06.048

**Published:** 2018-07-17

**Authors:** Emma L. Poole, Verity G. Kew, Jonathan C.H. Lau, Matthew J. Murray, Thomas Stamminger, John H. Sinclair, Matthew B. Reeves

**Affiliations:** 1 Department of Medicine, University of Cambridge, Addenbrooke’s Hospital, Cambridge CB2 2QQ, UK; 2 Institute of Immunity & Transplantation, University College London, Royal Free Campus, London NW3 2PF, UK; 3 Institute for Virology, Ulm University, Ulm 89081, Germany

**Keywords:** cytomegalovirus, latency, deSUMOylase, PML, reactivation

## Abstract

A subset of viral genes is required for the long-term latent infection of hematopoietic cells by human cytomegalovirus (HCMV). Here, we show that a latency-associated gene product (LUNA) promotes the disruption of cellular PML bodies during latency. Mutation and inhibitor studies reveal that LUNA encodes a deSUMOylase activity responsible for this disruption. Specifically, LUNA encodes a conserved Asp-Cys-Gly motif common to all deSUMOylases. Importantly, mutation of the putative catalytic cysteine is sufficient to reverse LUNA-mediated PML dispersal and markedly reduces the efficiency of viral reactivation. The depletion of PML from cells is sufficient to rescue the reactivation of the LUNA-deficient viruses, arguing that targeting PML is an important biological role of LUNA. Finally, we demonstrate that reactivation of naturally latent HCMV is blocked by deSUMOylase inhibitors. Thus, latent HCMV primes the cellular environment for efficient reactivation via the activity of a virally encoded deSUMOylase.

## Highlights

• Latent HCMV expresses a deSUMOylase (LUNA)
• LUNA promotes PML deSUMOylation and dispersal of ND10 bodies
• Targeting PML primes the cell for more efficient viral reactivation
• Isopeptidase inhibitors block viral reactivation

## Graphical Abstract

**Figure:**
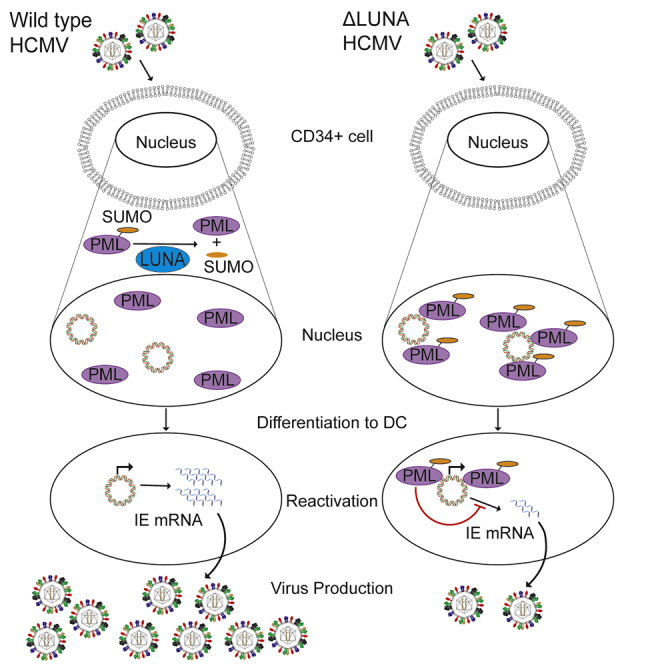

## Teaser

Poole et al. provide evidence that human cytomegalovirus removes anti-viral ND10 structures during latency. This serves to prime a cellular environment that can support reactivation as latently infected cells differentiate. This is achieved via the activity of a viral deSUMOylase that targets SUMOylated PML, an essential component required for ND10 formation.

## Introduction

Human cytomegalovirus (HCMV) establishes a lifelong latent infection of the hematopoietic CD34^+^ cell population resident in the bone marrow and persists in the cells of the myelo-monocytic cell lineage. HCMV infection *in utero* (Dollard et al., 2007) as well as in immune-compromised or immune-suppressed patients (Legendre and Pascual, 2008, Limaye et al., 2008) is a major cause of morbidity with reactivating virus, providing a significant contribution to the onset and progression of disease. As such, understanding the mechanisms that control latency, persistence, and reactivation of HCMV is of paramount importance in order to reduce the disease burden associated with this persistent human pathogen.

Studies from a number of laboratories now point toward a model of reactivation that argues that multiple events are required for the virus to exit latency. These events involve a range of viral and cellular functions that control viral gene expression, particularly, major immediate-early (MIE) gene expression (Dupont and Reeves, 2016). Supporting this view, reactivation studies of HCMV in myeloid progenitor cells isolated from naturally latent seropositive donors have shown that cellular differentiation to macrophages or dendritic cells, chromatin regulation of viral gene expression, inflammation, and associated cellular signaling are all important for HCMV reactivation to occur (Hahn et al., 1998, Hargett and Shenk, 2010, Reeves and Compton, 2011, Reeves et al., 2005b, Reeves and Sinclair, 2013, Söderberg-Nauclér et al., 1997, Zhuravskaya et al., 1997).

SUMOylation is one of a number of post-translational modifications (PTMs) that affect protein function in the cell (Müller et al., 2001). Currently, three related SUMO moieties have been best characterized (SUMO-1, SUMO-2, and SUMO-3). Conjugation of either the monomeric (SUMO-1) or polymeric (SUMO-2/3) forms drives the functional heterogeneity associated with these PTMs. A paralog (SUMO-4) has also been reported, although this precursor has a proline substitution at glutamine residue 90 rendering it insensitive to maturation, thus preventing its covalent bonding to proteins (Owerbach et al., 2005). Additionally, the report of SUMO-5 suggests greater diversity than first thought (Liang et al., 2016). In eukaryotic cells, SUMO de-conjugation requires isopeptidase activity, which, classically, is driven by six mammalian enzymes termed sentrin proteases (SENPs) (Hannoun et al., 2010), although the identification of two new classes of SUMO proteases suggests greater diversity than originally reported (Hickey et al., 2012). All SUMO proteases are cysteine proteases. Consequently, mutation of the catalytic cysteine residue results in a catalytically dead protein (Xu et al., 2006). Because all SENPs possess the necessary catalytic domain in their C terminus, it is argued that substrate specificity is achieved via specific sub-cellular localization, which is attributed to sequences encoded in the N terminus of the protein (Hannoun et al., 2010). To date, a number of SUMOylated cellular proteins have been identified, and they are predominantly proteins that function in the nucleus. As such, SUMOylation has been shown to affect chromatin structure, gene transcription, and DNA repair and, in some instances, may antagonize ubiquitin function through competition for shared lysine residues in target proteins (Hickey et al., 2012).

A pivotal cellular protein in the host response to viral infection is PML (Everett and Chelbi-Alix, 2007). PML is an interferon-inducible TRIM protein that can restrict viral infection (Everett and Chelbi-Alix, 2007, Kalejta, 2008, Tavalai and Stamminger, 2011). In the context of herpes virus infection, this is linked with the ability of PML to form sub-nuclear structures called PML bodies or ND10 (Everett and Chelbi-Alix, 2007). A number of cellular proteins with anti-viral activity (e.g., hDaxx, Sp100, ATRX, chromatin-modifying enzymes) accumulate here (Lukashchuk et al., 2008, Murphy et al., 2002, Saffert and Kalejta, 2006, Tavalai et al., 2011, Woodhall et al., 2006) preventing the initiation of viral IE gene expression. The formation of PML bodies is dependent on PML SUMOlyation (Ishov et al., 1999, Lallemand-Breitenbach and de Thé, 2010). Importantly, although not all PML is SUMOylated in PML bodies, the presence of SUMOylated PML is required for their formation (Lallemand-Breitenbach and de Thé, 2010). Consistent with this, overexpression of cellular SENPs promotes the dispersal of PML bodies (Cheng and Kao, 2013).

The anti-viral nature of PML and ND10 bodies make them important targets for virally encoded antagonists. The herpes simplex virus (HSV) protein, ICP0, has been demonstrated to operate as a SUMO-targeting ubiquitin ligase (STUbL) promoting the degradation of many SUMOylated proteins, including PML (Boutell et al., 2011). However, its homolog in HCMV (IE72) does not act as a STUbL (Scherer et al., 2013). Instead, IE72 promotes PML deSUMOylation but not degradation in lytic infection (Lee et al., 2004). As such, the dispersal of ND10 bodies along with the targeted degradation of specific components by virion tegument proteins such as pp71 represent important events in the initiation of HCMV lytic gene expression (Tavalai and Stamminger, 2011).

In this study, we have investigated the function of a viral gene product expressed during both latent and lytic infection, LUNA (Bego et al., 2005, Reeves and Sinclair, 2010). Previous data have implicated a requirement for LUNA during reactivation in monocytes (Keyes et al., 2012b), but the mechanism remained unclear. Here, we report that LUNA is a cysteine protease with deSUMOylase activity. LUNA transfection promoted dispersal of PML bodies, and furthermore, latent HCMV infection promoted PML body dispersal in a LUNA-dependent manner. Although LUNA was not required for latent carriage, a profound defect in reactivation was observed in both the LUNA-null virus and the virus expressing a catalytically dead version of LUNA. Importantly, depletion of PML from cells rescues reactivation of the LUNA defective viruses, arguing that deSUMOylation of PML by LUNA is biologically important for reactivation. Taken together, these data suggest that LUNA, via a deSUMOylase activity, eliminates potentially anti-viral PML body structures from the cell during latency. We propose that this serves as a mechanism to optimize the cellular environment for efficient reactivation by removing a potential inhibitor of lytic infection independently of tegument or IE gene function.

## Results

### LUNA Expression Is Important for HCMV Reactivation from CD34^+^-Derived Dendritic Cells

**Figure 1:**
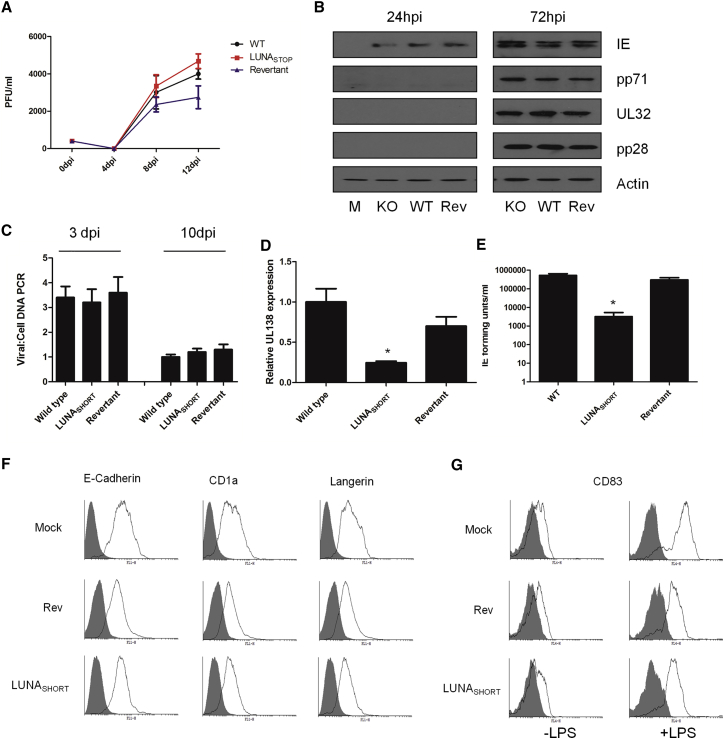
LUNA Is Required for Efficient Reactivation in CD34^+^-Derived DCs (A) HFFs were infected at an MOI of 0.1 with Merlin, LUNA_SHORT_, or revertant virus, and the growth was measured over 14 days by titration of supernatants for infectious virus production every 2 days. (B) Viral gene expression was also assessed by western blotting of mock infected cells (M) or cells infected with LUNA_SHORT_ (KO), wild-type (WT), or revertant (Rev) virus at 24 and 72 hrs post-infection (hpi). (C) CD34^+^ cells infected with WT, revertant, or LUNA_SHORT_ were analyzed using qPCR for viral and cellular DNA levels at 3 dpi and then at 10 dpi after differentiation to an immature CD34^+^-derived DC phenotype. (D) RNA isolated from WT revertant or LUNA_SHORT_ infected CD34^+^ cells at 7 dpi were analyzed for UL138 gene expression using qRT-PCR. All samples were normalized to GAPDH and then expressed relative to WT virus. (E) CD34^+^ cells infected for 3 days to establish latency with WT, revertant, and LUNA_SHORT_ viruses were differentiated and matured into CD34^+^-derived DCs to induce reactivation and then co-cultured with fibroblasts. At 15 days, co-cultures were harvested and assayed for infectious virus production (IE forming units) on fresh fibroblasts. In (A) and (C)–(E), data are mean ± SD and represent triplicate analyses performed in two independent experiments. ^∗^p < 0.05. (F) Cell surface phenotyping for E-cadherin, CD1a, and CD207/Langerin expression was performed on DCs derived from CD34^+^ cells latently infected with mock, revertant, or LUNA_SHORT_ virus. Isotype (shaded) and specific antibody (open) staining are shown. (G) Cell surface phenotyping for CD83 expression was performed on immature (−LPS) and mature (+LPS) DCs derived from CD34^+^ cells latently infected with mock, revertant, or LUNA_SHORT_ virus. Isotype (shaded) and specific antibody (open) staining are shown.

To address the function of LUNA during latent infection, we generated a virus that was mutated to prevent LUNA expression but did not disrupt the UL82 gene on the cDNA strand (LUNA_SHORT_). First, the growth of the virus was assayed in both lytic and latent infection. Consistent with previous data (Keyes et al., 2012b), disruption of LUNA had little impact on the growth of Merlin HCMV in fibroblasts at a low MOI (Figure 1A), nor the expression of viral gene products including pp71 (Figure 1B). We next characterized the virus during latent infection of CD34^+^ cells (Figures 1C–1E). CD34^+^ cells were latently infected with wild-type Merlin virus, LUNA_SHORT_ virus, or a revertant virus and then assayed for DNA carriage between 3–10 days post-infection (dpi) in the absence of any cytokine stimulation (Figure 1C). Although the ratio of viral to cellular DNA did show a decrease over time for all viruses, disruption of LUNA protein did not appear to have any differential impact on the carriage of HCMV genomes in CD34^+^ cells compared with wild-type virus (Figure 1C). However, we did note a 5-fold decrease in the level of UL138 expression during latent infection with LUNA_SHORT_ virus compared with wild-type (Figure 1D), which could not be explained by differential genome carriage alone (Figure 1C). Consistent with a previous study (Keyes et al., 2012b), the most profound effect of LUNA protein disruption was during reactivation (Figure 1E). In CD34^+^ cells, differentiated to dendritic cells (DCs) to promote reactivation of infectious virus, we observed a substantial defect in the reactivation of the LUNA_SHORT_ virus (Figure 1E). This defect in reactivation was not associated with a failure of LUNA_SHORT_ virus-infected CD34^+^ cells to differentiate into Langerhans-like DCs (Figure 1F) nor mature in response to lipopolysaccharide (LPS), as evidenced by an induction of CD83 (Figure 1G).

### LUNA Encodes an Isopeptidase that Disrupts PML Bodies upon Transfection and in Latent Infection

**Figure 2:**
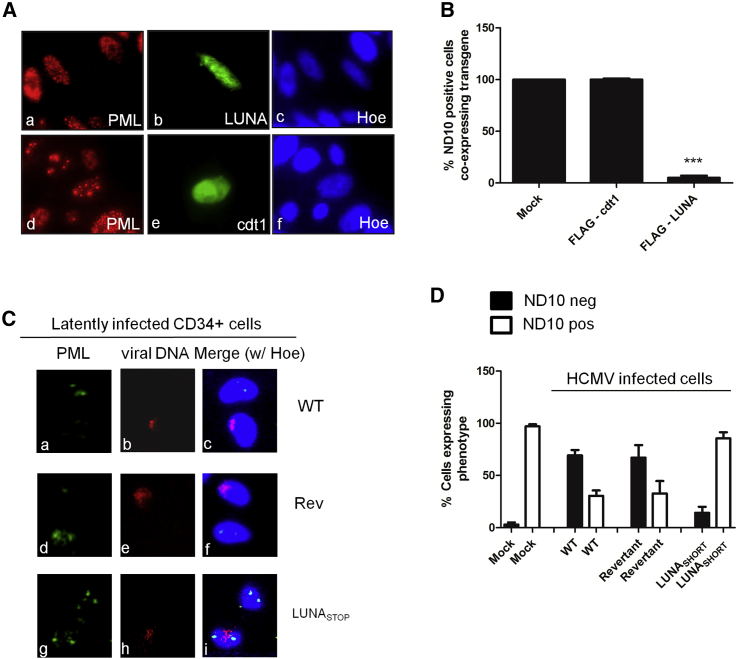
LUNA Transfection and Latent Infection Promotes Disruption of ND10 Structures (A) N-terminal FLAG-tagged LUNA (a–c) or FLAG-tagged cellular protein cdt1 (d–f) were transfected into fibroblasts, and cells were co-stained 48 hr later for FLAG (b and e) and PML (a and d). (B) Enumeration of multiple transfection analyses for ND10 disruption Data are mean ± SD and represent triplicate analyses performed in eight independent experiments. ^∗∗∗^p < 0.001. (C) CD34^+^ cells latently infected with WT Merlin (a–c; WT), revertant virus (d–f; Rev), or the LUNA translation mutant virus (g–i; LUNA_SHORT_) were stained for PML (a, d, and g) or viral genome (b, e, and h) 7 dpi. (D) The number of infected ND10 positive or negative CD34^+^ cells was scored from ten fields of view at 7 dpi with mock, Merlin (WT), revertant (Rev), and LUNA mutant (LUNA_SHORT_). See also Figure S1.

To interrogate LUNA function in more detail, we took a reductionist approach and first asked where LUNA protein localized upon transfection. A previous study hypothesized that LUNA could antagonize the activity of the viral tegument protein pp71 against ND10 components (Bego et al., 2005). Consequently, we transfected fibroblast cells and co-stained them for LUNA and PML (an essential component of ND10) to determine whether the LUNA protein localized to these nuclear structures (Figure 2). The transfected LUNA protein was predominantly nuclear but did not co-localize with PML. Surprisingly, LUNA promoted the disruption of ND10 bodies (Figure 2A). Quantification of multiple experiments revealed that LUNA-positive cells rarely contained normal ND10 bodies (Figure 2B). This observation was not a non-specific effect associated with the overexpression of a nuclear protein, as cells transfected with cdt1 remained ND10 positive (Figures 2A and 2B). LUNA-mediated disruption of ND10 bodies was also evident in lentivirus-transduced U937 myeloid cells, suggesting that this could occur during latent infection (Figure S1). Consistent with this, the same disrupted ND10 body phenotype was also observed in long-term latently infected CD34^+^ cells identified by genome fluorescence *in situ* hybridization (FISH) (Figures 2C and 2D). In contrast, analysis of CD34^+^ cells latently infected with LUNA_SHORT_ virus revealed that the majority of cells containing viral genome remained ND10 body positive (Figures 2C and 2D).

**Figure 3:**
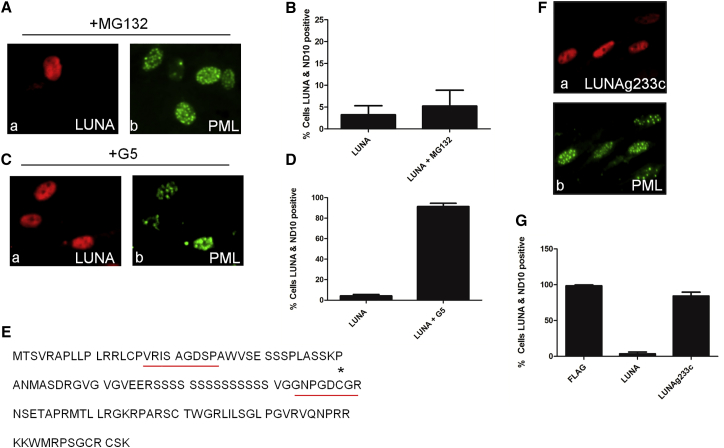
LUNA Encodes a Cysteine Protease Sensitive to Isopeptidase Inhibitors (A and B) HFFs transfected with LUNA were incubated with MG132 and analyzed for FLAG (a) and PML expression (b) 24 hr post-transfection (A) and number of ND10- and LUNA-positive cells enumerated (B). Data are mean ± SD and represent triplicate analyses performed in three independent experiments. (C and D) N-terminal FLAG tagged LUNA (LUNA) transfected into fibroblasts were incubated with isopeptidase inhibitor, G5, and stained for FLAG (a) and PML (b) expression 48 hr later (C) and number of ND10- and LUNA-positive cells enumerated (D). Data are mean ± SD and represent triplicate analyses performed in three independent experiments. (E) Identification of the two conserved motifs in LUNA (red box). Asterisk denotes catalytic cysteine. (F and G) N-terminal FLAG tagged catalytic dead mutant of LUNA (LUNAg233c) was transfected into fibroblasts and stained for FLAG (a) and PML (b) expression 48 hr later (F) and the number of ND10- and LUNA-positive cells enumerated (G). Data are mean ± SD and represent triplicate analyses performed in three independent experiments. See also Figures S2 and S3.

To investigate the mechanistic basis of the LUNA phenotype, we initially transfected fragments of LUNA into cells (Figures S2A and S2B) and measured their ability to disrupt ND10 bodies. This approach suggested that the core ND10-disrupting activity of LUNA resided in the C-terminal domain (Figures S2A and S2B). A bioinformatics analysis of amino acids 70–133 of LUNA for motifs (InterPro; European Molecular Biology Laboratory–European Bioinformatics Institute [EMBL-EBI]) revealed minimal sequence homology with known cellular proteins but reported an identity with ubiquitin-like modifying enzymes. It has been previously demonstrated that the dispersal of ND10 bodies by the HSV protein, ICP0, is dependent on an E3 ligase function and thus is can be inhibited by the proteasome inhibitor MG132 (Everett et al., 1998). However, the addition of MG132 did not prevent ND10 body dispersal (Figures 3A and 3B), suggesting that this ND10 disruption activity of LUNA was independent of the proteosome and any E3 ubiquitin ligase activity.

The failure to observe any effects with MG132 and the predicted homology with ubiquitin-like modifying enzymes led us to question whether LUNA’s role during latency could involve the closely related PTM of proteins by SUMOylation. DeSUMOylases cleave isopeptide bonds to remove SUMO moieties to proteins. Addition of an isopeptidase inhibitor, G5, clearly inhibited LUNA-mediated disruption of ND10 bodies (Figures 3C and 3D). Interestingly, G5 had no impact on IE72-mediated disruption of ND10 bodies during virus lytic infection, suggesting a LUNA-specific effect (Figure S3), suggesting that the LUNA-mediated disruption required an isopeptidase activity. Scanning of the C terminus of the LUNA amino acid sequence revealed a candidate motif (NxxDCG) containing a putative catalytic cysteine flanked by aspartic acid and glycine residues (Figure 3E) (Kim et al., 2000). Additionally, LUNA encoded a characteristic IxxxDS motif also observed in eukaryotic deSUMOylases (Figure 3E). Many studies of eukaryotic deSUMOylases (SENPs) have relied on the mutation of this cysteine to a serine or alanine to generate catalytically dead isopeptidases (Hickey et al., 2012). Thus, we generated a cysteine-serine mutation in LUNA (LUNAg233c). The disruption of ND10 bodies was abrogated in cells transfected with LUNAg233c (Figures 3F and 3G) providing further support for LUNA encoding a deSUMOylase activity.

**Figure 4:**
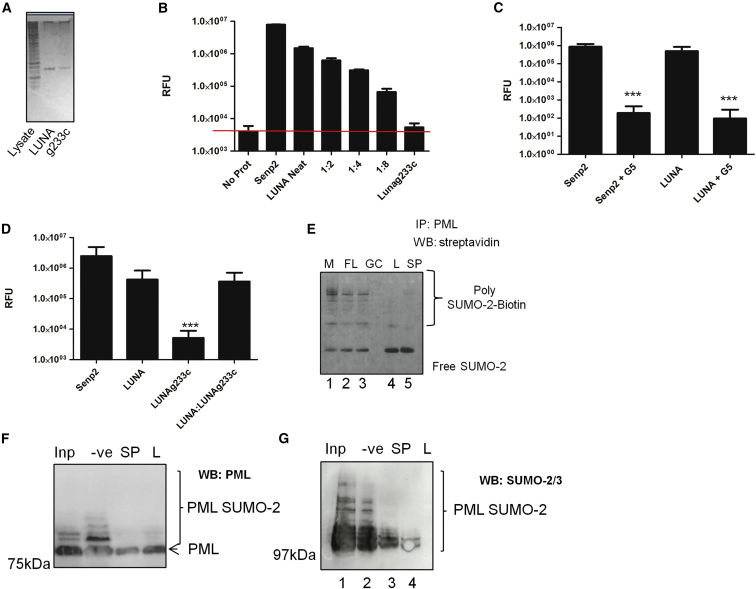
LUNA Encodes an Isopeptidase Activity that DeSUMOylates PML *In Vitro* (A) Silver stain of purified LUNA (LUN) and LUNA g233c proteins (g233c) used for downstream analyses. A cell lysate (lysate) is shown for contrast. (B) Cleavage of SUMO-3 was measured by fluorescent activity of a substrate in a reporter assay. Substrate (No Prot) was incubated with SENP2_CD_, or 2-fold dilutions of LUNA (1:2–1:8) or LUNAg233c (g233c) and fluorescent intensity measured. Red line indicates background signal (No Prot). (C) The same assay was used as above except that prior to assay, the SENP2_CD_ or LUNA protein was incubated with DMSO or 1 μM G5 for 30 min. (D) Senp2, LUNA, LUNAg233c, or equimolar concentrations of LUNA:LUNAg233c were assessed for isopeptidase activity in the reporter assay. Data are mean ± SD and represent triplicate analyses performed in two independent experiments (C and D). ^∗∗∗^p < 0.001. (E) PML immuno-precipitated from cell extracts was subsequently incubated with E1 and E2 enzymes and biotin labeled poly-SUMO-2. Following SUMOylation, the SUMOlyated PML was subsequently incubated with buffer (M) or TnT reactions producing FLAG (FL), LUNAg233c (GC), or LUNA (L) or with a recombinant SENP2_CD_ (SP) enzyme for 30 min. Samples were analyzed by western blot using streptavidin conjugated-HRP. (F and G) Bacterially expressed PML IV was SUMOylated *in vitro* with poly-SUMO-2 chains (Inp, track 1), and aliquots were incubated with buffer (−ve, track 2), SENP2_CD_ (SP, track 3), or bacterially expressed LUNA (L, track 4) for 30 min, and then the same samples were analyzed using western blot with PML (F) and poly-SUMO-2 (G) antibodies. See also Figure S4.

To test biochemically for isopeptidase activity, we used an ELISA-based approach. We generated purified recombinant LUNA and LUNA g233c proteins (Figure 4A) and tested them for isopeptidase activity (Figures 4B–4D). The data show that LUNA does have isopeptidase activity (Figure 4B) and that the activity of LUNA (and the positive control SENP2) was sensitive to the G5 isopeptidase inhibitor (Figure 4C). Furthermore, equivalent amounts of LUNAg223c exhibited no activity in the same assay (Figure 4D). Finally, a 1:1 mixture of LUNA and LUNAg233c had no impact on the activity of LUNA in this assay, arguing against the possibility that the lack of isopeptidase activity associated with LUNAg233c was due to an unidentified contaminant in the LUNAg233c protein preparation that inhibited isopeptidase activity (Figure 4D).

### LUNA Displays Evidence of Isopeptidase, but Not Endopeptidase, Activity against SUMO Isoforms

Eukaryotic SENPs possess both SUMO chain editing (isopeptidase) and SUMO processing activity (endopeptidase). When a pre-SUMO-1 recombinant protein was purified LUNA protein, we could find no evidence for endopeptidase activity (Figure S4). Thus we focused on isopeptidase activity testing whether poly-SUMO-2 chains were degraded by LUNA. To do this, we conjugated poly-SUMO-2 chains to PML *in vitro* and then incubated them control SENP2_CD_ or LUNA protein (Figure 4E). In this assay, the processing of SUMO-2 chains bound to PML was evident with both SENP2_CD_ and LUNA (lanes 4 and 5). In contrast, the elimination of the putative catalytic cysteine (LUNAg233c; lane 3) blocked this activity. Taken together these data suggested that LUNA, unlike most eukaryotic SENPs (Hickey et al., 2012), possessed isopeptidase but not endopeptidase activity.

We next investigated whether bacterially expressed LUNA could de-conjugate poly-SUMO chains from PML. Recombinant PML was subjected to a SUMOylation reaction *in vitro*. Consistent with PML SUMOylation, multiple high-molecular weight (MW) bands of PML protein were observed (Figure 4F, lane 1) which were confirmed to be SUMOylated PML by blotting the same samples with a SUMO-2/3 antibody (Figure 4G, lane 1). The SUMO-modified PML was then incubated with buffer alone, SENP2_CD_, or LUNA (Figures 4F and 4G, lanes 2–4). The data show that incubation of SUMOylated PML with SENP2_CD_ or LUNA resulted in a loss of high-MW PML species (Figure 4F, lanes 3 and 4) and, specifically, because of a loss of SUMOylated PML protein (Figure 4G, lanes 3 and 4). Thus, SENP2 and LUNA promote the deSUMOylation of SUMOylated PML *in vitro* but not the degradation of PML itself.

### Isopeptidase Activity Is Required for HCMV Reactivation from Latency

**Figure 5:**
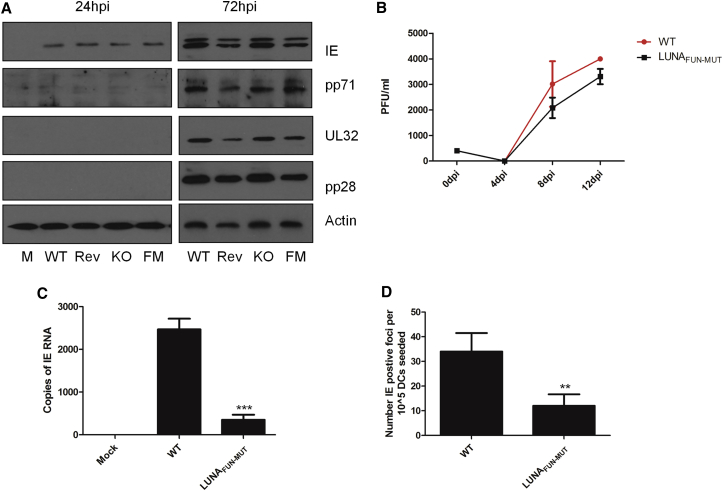
Mutation of the Putative Catalytic Site of LUNA Abrogates HCMV Reactivation from Latency (A) HFFs were infected at an MOI of 0.1 with WT Merlin, LUNA catalytic dead mutant (LUNA_FUN-MUT_), or WT virus and the growth measured over 14 days. Supernatants were titered for infectious virus production every 2 days. (B) Western blotting on mock (M), LUNA_SHORT_ (KO), WT, revertant (Rev), or LUNA functional mutant (FM) at 24 and 72 hpi for viral protein expression. (C and D) CD34^+^ cells latently infected with WT or a catalytic dead mutant of LUNA (LUNA_FUN-MUT_) were differentiated to mature DCs. Induction of IE gene expression (C) or reactivation of virus by infectious center formation in co-cultures (D) was quantified by qRT-PCR or immuno-staining, respectively. Data are mean ± SD and represent triplicate analyses performed in three independent experiments. ^∗∗^p < 0.01 and ^∗∗∗^p < 0.001.

Thus far, our analyses were consistent with LUNA expression being important for HCMV reactivation in CD34^+^-derived DCs and that LUNA encoded a putative deSUMOylase activity. Thus, we next sought to determine whether it was the deSUMOylase activity that underpinned LUNA’s role in reactivation. A virus carrying a point mutant in LUNA that eliminated the catalytic cysteine was generated (LUNA_FUN-MUT_). Analysis of viral protein expression revealed no major defects in their accumulation and, consistent with this, the growth kinetics of the mutant were similar to the wild-type (WT) virus (Figures 5A and 5B). We next asked whether this mutation in LUNA had any impact on virus reactivation. The induction of IE gene expression from latency was markedly impaired in the LUNA_FUN-MUT_-infected cells compared with WT (Figure 5C). This defect in IE transcription translated into a reactivation deficit. Using an infectious foci assay, whereby DCs are co-cultured with human foreskin fibroblasts (HFFs) and the HFF monolayer stained for IE gene expression to detect evidence of viral reactivation, we could clearly see an impact on viral reactivation in the LUNA_FUN-MUT_ (Figure 5D).

**Figure 6:**
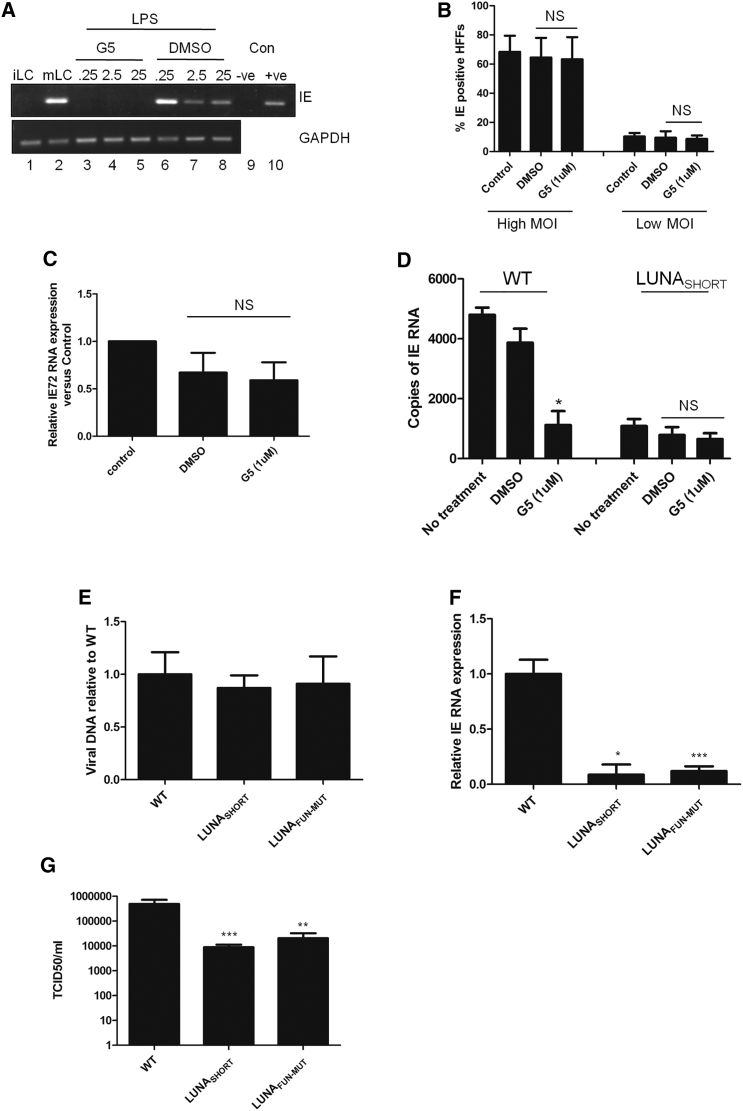
LUNA Isopeptidase Activity Is Required for HCMV Reactivation (A) CD34^+^ cells isolated from a healthy seropositive donor were cultured to immature DCs (iLC; lane 1) and then incubated with LPS to promote full virus reactivation (mLC; lanes 2–8). Prior to the addition of LPS, cells were incubated with mock (2), G5 isopeptidase inhibitor (25–0.25 μM; 3–5), or DMSO solvent (6–8) for 2 hr. Reactivation was measured using RT-PCR for IE72 expression on RNA isolated from cells 24 hr post-addition of LPS. Water (−ve) and cDNA from infected fibroblasts (+ve) served as PCR controls (lanes 9 and 10). (B) HFFs were incubated with 1 μM G5 and infected with HCMV at an MOI of 1 (high MOI) or 0.1 (low MOI) and then analyzed by immunofluorescence (IF) at 8 hr post-infection for IE gene expression, and percentage infection was calculated. (C) A qRT-PCR analysis for IE gene expression was performed on low-MOI infected HFF cells as described in (B). (D) CD34^+^ cells latently infected with WT or the LUNA protein disruption virus (LUNA_SHORT_) were differentiated to immature DCs and then either incubated with DMSO or G5 prior to stimulation with LPS to fully reactivate virus. Twenty-four hours post-infection RNA was isolated and analyzed using IE qRT-PCR and quantified against a standard curve. Data are mean ± SD and represent triplicate analyses performed in three independent experiments. ^∗^p < 0.05; NS, not significant. (E–G) CD34^+^ cells from two donors were infected with Merlin, LUNA_SHORT_, or LUNA_FUN-MUT_. Seven dpi cells were differentiated to immature DCs and analyzed using qPCR for viral genome carriage with viral genomes expressed relative to cellular DNA control (E). Alternatively, immature CD34^+^-derived DCs were incubated with LPS and then either analyzed for IE RNA expression by qRT-PCR (F) or co-cultured with HFFs and assayed for infectious virus production (G). Data are mean ± SD and represent triplicate analyses performed in two independent experiments. ^∗^p < 0.05, ^∗∗^p < 0.01, and ^∗∗∗^p < 0.001; NS, not significant (n = 2). See also Figure S5.

These data predicted that HCMV reactivation, or at least the de-repression and induction of IE gene expression as latently infected cells reactivated, could be sensitive to the activity of isopeptidase inhibitors. Strong evidence in support of this was a study of naturally latent CD34^+^ cells isolated from seropositive donors. Differentiation to DCs and stimulation with LPS promotes IE gene expression, an event inhibited by the G5 isopeptidase inhibitor (Figure 6A). We saw no effect on DC maturation in the presence of G5, arguing against this being a non-specific effect on cellular differentiation, an important event for HCMV reactivation (Figure S5). Furthermore, the effects of G5 were restricted to reactivation, as little impact on infection and IE gene expression during lytic infection at high or low MOIs was observed (Figures 6B and 6C). Thus, these data pointed toward an isopeptidase activity being important during the very early stages of viral reactivation (i.e., during the reactivation of IE gene expression from latency).

Clearly, at this stage, we could not rule out the possibility that inhibition of cellular isopeptidases was responsible for the lack of IE gene expression detected in the reactivating DCs. To address this, we took advantage of the fact that although reactivation of the LUNA_SHORT_ is severely abrogated, it does show detectable levels of reactivation (Figure 1). Consequently, we asked whether the low but detectable level of reactivation of LUNA_SHORT_ virus could be further abrogated by the G5 isopeptidase inhibitor (Figure 6D), on the basis that if LUNA were the predominant isopeptidase activity required for this early event in reactivation, then the low level of reactivation of the LUNA_SHORT_ virus would be largely resistant to further inhibitory effects of the compound. As before, we observed that the LUNA_SHORT_ virus reactivated IE RNA expression far less efficiently than WT virus (compare WT and LUNA_SHORT_ with no treatment: Figure 6D, bars 1–3). However, although treatment with G5 inhibitor resulted in a profound effect on IE reactivation of WT virus, G5 treatment of cells latently infected with LUNA_SHORT_ virus did not have a significant impact on IE expression (Figure 6D).

To investigate the contribution of the LUNA encoded deSUMOylase activity on the overall impact of LUNA on HCMV reactivation, we performed a direct comparison of the LUNA mutants with the Merlin parent virus upon reactivation (Figures 6E–6G). Latently, infected CD34^+^ cells from two donors were subsequently differentiated to immature DCs at 7 dpi. A qPCR analysis for viral genomes at this stage revealed that only minor differences in genome copy number were evident compared with WT Merlin in the immature DCs (Figure 6E). We then stimulated immature DCs with LPS and analyzed IE RNA expression. A clear defect in the induction of IE gene expression was observed, and furthermore, it was similarly impaired in both LUNA mutant viruses, although the disruption of LUNA protein expression (LUNA_SHORT_) appeared to have a slightly greater impact (Figure 6F). Crucially, a comparison of reactivation of infectious virus in co-culture assays revealed that although both mutant viruses exhibited a defect in reactivation (1.5–2 log reduction), the disruption of LUNA protein expression had a markedly greater impact (Figure 6G), approaching a 0.5 log greater defect compared with the catalytic mutant (LUNA_FUN-MUT_).

### Depletion of PML Rescues the Reactivation of LUNA-Deficient Viruses

**Figure 7:**
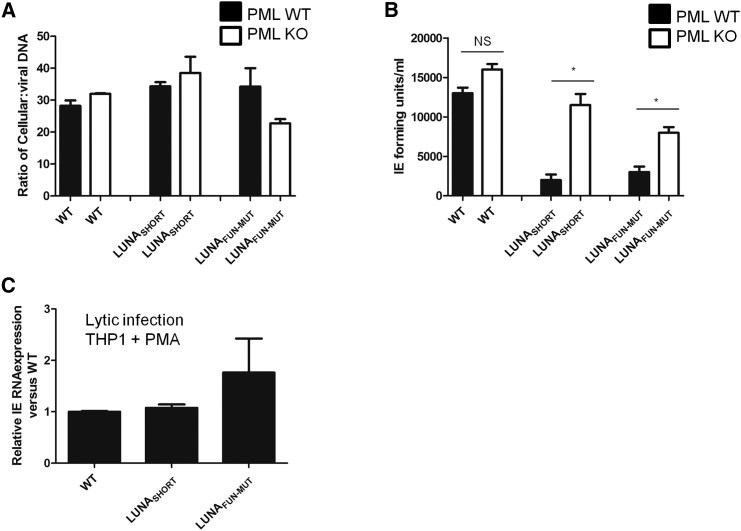
Depletion of PML Rescues the Reactivation of LUNA-Deficient Viruses (A and B) WT (black bar) or PML knockout (gray bar) cells were infected with Merlin (WT), LUNA protein disruption (LUNA_SHORT_), or LUNA catalytic dead mutant (LUNA_FUN-MUT_) to establish a latent infection. Prior to stimulation of reactivation, total DNA was quantified using qPCR for viral and cellular genomes and expressed as a ratio (A). Following stimulation, latent THP1 cells were incubated with HFFs for 12 days and the supernatants analyzed for production of infectious virus using in IE forming units assay (B). Data are mean ± SD and represent triplicate analyses performed in three independent experiments. ^∗^p < 0.05; NS, not significant. (C) THP1 cells were differentiated with PMA and then infected with Merlin (WT), LUNA protein disruption (LUNA_SHORT_), or LUNA catalytic dead mutant (LUNA_FUN-MUT_). RNA was isolated 16 hpi and analyzed using qRT-PCR for IE and 18S expression, and then fold difference from WT was calculated using the 2^−ΔΔCT^ method. Data are mean ± SD and represent triplicate analyses performed in two independent experiments. See also Figure S6.

Although the activity of LUNA against PML helped identify the enzymatic function of LUNA, we wanted to assess if targeting PML for deSUMOylation was biologically important for reactivation. To do this, we used a THP1 latency model (Arcangeletti et al., 2016, Keyes et al., 2012a, Lau et al., 2016) to investigate whether deletion of PML from myeloid cells affected reactivation. First, WT or PML knockout (KO) THP1 cells were infected with HCMV, and the carriage of viral genomes was analyzed (Figure 7A). No differences were observed if either cell type was infected with Merlin or the two mutant viruses, suggesting no overt impact on carriage. Having established no impact on genome carriage, THP1 cells were stimulated with PMA to reactivate the latent infection (Figure 7B). The data show that the reactivation of the LUNA defective HCMVs was impaired in the WT THP1 cells compared with Merlin WT virus. However, reactivation in cells deleted for PML clearly rescued reactivation of the LUNA defective viruses to levels approaching WT HCMV (Figure 7B). It was also noted that a minor enhancement of the reactivation of WT HCMV was observed, although this did not reach statistical significance (Figure 7B). Finally, we demonstrate that the effect on IE in THP1 cells is specific for reactivation, as the WT and mutant viruses displayed no differential capacity to establish lytic infections of control and PML KO THP1 cells (Figures 7C and S6).

## Discussion

The reactivation of HCMV is a complex interplay of host and cellular factors that will cumulatively contribute to the process. In this study, we have provided evidence that a latent viral gene product, LUNA, encodes a deSUMOylase activity that increases the efficiency of viral reactivation. Our data suggest that this viral activity is important during the very early stages of HCMV reactivation, leading to the robust induction of IE gene expression, which will have downstream consequences on viral reactivation. Thus, we propose a model whereby a latent gene product primes the cellular environment for reactivation through the disabling of an important anti-viral function (ND10) known to be inhibitory to lytic infection. We cannot rule out, nor do we think it is likely, that the only isopeptidase activity important for the complete reactivation of infectious virus is virally encoded, as SUMOylation and ubiquitination are important regulators of many cellular processes that could regulate viral replication.

Although we argue that the most prominent role of LUNA is during the early stages of reactivation, we note that an impact on UL138 gene expression was observed. Because it has previously been shown that UL138 expression is important for HCMV latency (Goodrum et al., 2007) the effects we observe could be linked with reduced UL138 expression in the LUNA mutant virus-infected cells. However, we found no evidence that the LUNA mutant viruses lytically infected CD34^+^ cells (as has been proposed for the UL138 deletion virus) and thus does not display a major characteristic of the UL138 deletion viruses. We speculate that this is because UL138 expression, although lower, is still sufficient to prevent lytic infection in the CD34^+^ cells.

The identification of a functional interaction between LUNA and PML, a critical component of ND10 bodies, resulting in the dispersal of ND10 in latently infected cells at first seemed counterintuitive. ND10 bodies repress HCMV IE gene expression, and ND10 disruption is known to be required for robust HCMV IE gene expression, a theme common to many viruses (Everett and Chelbi-Alix, 2007, Geoffroy and Chelbi-Alix, 2011, Tavalai and Stamminger, 2011). Put simply, why is IE gene expression not initiated in the latently infected cells if ND10 bodies have been removed? The simplest explanation is that ND10 bodies are not required for long-term silencing of the major IE promoter (MIEP) during latency (Wagenknecht et al., 2015). Instead, high levels of transcriptional repressors of the MIEP present in undifferentiated myeloid cells promote the formation of a repressive higher order chromatin structure around the MIEP (Sinclair and Sissons, 2006). These effects are likely augmented by other viral functions that inhibit the MIEP (Lee et al., 2015, Rossetto et al., 2013) as well as an absence of viral MIEP activators such as pp71 (Saffert et al., 2010). Reactivation, then, is likely triggered by a differentiation-dependent increase in the binding of transcriptional activators, a concomitant decrease in repressor binding, and the accompanying modification of chromatin around the MIEP that supports viral IE gene expression. The factors that drive this are probably a concert of signaling pathways which become active during cellular differentiation and, relevant to disease settings, are exacerbated by inflammation (Hahn et al., 1998, Hargett and Shenk, 2010, Humar et al., 1999, Kew et al., 2014, Reeves and Compton, 2011, Söderberg-Nauclér et al., 1997).

Reactivation is essentially the re-entry of the virus into the lytic life cycle, which is dependent on efficient major IE expression from the viral MIEP. Thus it can be hypothesized that, upon initiation of IE transcription as a result of myeloid cell differentiation, the MIEP regains sensitivity to the repressive effects of ND10 bodies (Groves et al., 2009, Lukashchuk et al., 2008, Tavalai et al., 2006). Thus the virus encounters a major problem: on exit from latency it must now neutralize the anti-viral function of ND10 bodies in the absence of IE72 and pp71 expression to ensure reactivation proceeds efficiently. For these reasons, we speculate that the removal of ND10 bodies by LUNA during latency eliminates a secondary restriction of full MIEP activity that would otherwise be encountered by reactivating virus if ND10 bodies were still present. Essentially, ND10 body disruption is not directly required to drive the virus to exit latency; ND10-mediated MIEP repression needs to be prevented once MIEP activity has been initiated by myeloid differentiation. Consistent with this, our data show that deletion of PML in THP-1 cells could significantly rescue the reactivation of the LUNA mutant viruses in these cells, suggesting that the activity of LUNA against PML is an important aspect of HCMV reactivation.

It is of course highly plausible that LUNA targets other cellular functions controlled by SUMOylation besides ND10 structures during reactivation, and we are currently addressing this. For instance, the regulation of chromatin structure and the activity of chromatin remodeling enzymes are subject to complex regulatory mechanisms including SUMOylation (Hickey et al., 2012). Similarly, it appears that the lack of LUNA expression in the LUNA_SHORT_ virus (compared with a mutation in the putative catalytic of LUNA domain alone (LUNA_FUN-MUT_) has a more overt effect on the reactivation of infectious virus. This could suggest that although the isopeptidase function of LUNA may be important during the initial stages of reactivation, additional LUNA functions, which are viral isopeptidase independent, also play a role in reactivation.

Another interesting aspect of our study is the observation that recombinant LUNA had isopeptidase activity but not processing or endopeptidase activity directed against pre-SUMO isoforms, which is in contrast to most cellular SENP proteins (Hickey et al., 2012). Whether these differences are linked with the limited sequence homology between LUNA and other cellular isopeptidases such as SENPs (e.g., the presence of a putative a catalytic dyad and not triad in LUNA) is unclear. However, the identification of a viral deSUMOylase with limited sequence homology to classical SENPs may be consistent with accumulating evidence that deSUMOylase function is expressed by an increasingly diverse range of proteins (Hickey et al., 2012) and also supportive of the concept that other viruses may also encode deSUMOylases that are similarly divergent from cellular SENPs. Finally, it is intriguing that LUNA does not encode a histidine residue to complete the catalytic triad associated with SENPs. Again, we note that a second class of deSUMOylating isopeptidases (DESIs) have been reported (Shin et al., 2012) to encode a catalytic dyad (Cys-His) and not a triad. Thus, whether LUNA represents a virally encoded divergent class of deSUMOylases remains to be determined.

Our data underscore the view that HCMV latency is an active process with latency-associated viral gene expression imparting a unique signature on the latently infected cell.

In part, this is mediated by the expression of virally encoded deSUMOylase activity that primes the latently infected cell for efficient viral reactivation. These data exemplify the emerging concept that latent viruses manipulate the cellular environment to ensure that efficient reactivation ensues when optimal conditions are met.

## Experimental Procedures

### Ethical Statement

All research describing studies on primary human material with HCMV were assessed and approved by the University College London (UCL) and Cambridge Local Research Ethics committees. Informed consent was received for the collection of leukapheresis products from granulocyte-colony-stimulating factor-mobilized patients and was performed in accordance with established guidelines for the handling and processing of said tissue by the UCL and Cambridge Local Research Ethics committees and the Cambridge Internal Review Board. Cells were harvested from healthy adult donors, and the decision to use tissue was not affected by gender and age (where known), as this was not important to the studies performed.

### Plasmids, Transfections, and Viruses

The LUNA coding region was excised from PET102UL82as (Bego et al., 2005) (BamHI/HindIII) and inserted pCMVTag2B, generating a N-terminal FLAG-LUNA. Site-directed mutagenesis introduced a guanine to cytosine nucleotide exchange at position 233 of the LUNA nucleotide sequence, replacing the cysteine for a serine residue (LUNAg233c). Fibroblasts (80% confluency) were transfected using Lipofectamine 2000 as described by manufacturer. Briefly, fibroblasts cultured in reduced serum (4 hr) were then incubated with Lipofectamine:plasmid DNA complex for 4 hr before recovery in DMEM-10. Analyses were performed 24–48 hr post-transfection.

BAC recombineering was used to generate a translation mutant in the Merlin backbone using previously described techniques (Stanton et al., 2010). Briefly, the cloning strategy for the LUNA_SHORT_ introduced a guanine-to-adenine exchange at nucleotide position 118965, mutating the tryptophan (TGG) to a STOP codon (TGA). Using this strategy, we were able to maintain the integrity of pp71 on the anti-parallel strand, as the codon change, ACC to ACA, was redundant for threonine. Mutation of the catalytic domain of the LUNA gene (termed LUNA_FUN-MUT_) exchanged a guanine-to-cytosine nucleotide exchange at position 233 of the LUNA nucleotide sequence (i.e., the plasmid mutation). This replaced the cysteine for a serine residue at nucleotide position 119197. Following sequence verification, the recombinant BAC viruses were then transfected into fibroblasts to reconstitute infectious virus. For these studies Merlin and mutants generated were propagated in adult retinal pigment epithelial cells or fibroblasts (Merlin and associated BAC were a kind gift of Gavin Wilkinson,).

### Fusion Proteins and SUMOylation Reactions

PML and LUNA fusion proteins were generated from BL21 bacteria transfected with PET102UL82as plasmid (Bego et al., 2005) or pGEX-PML (Everett et al., 1999) using previously published methods (Caswell et al., 1993). PML was SUMOylated *in vitro* using a SUMOLink kit (Active Motif). DeSUMOylation reactions were performed on aliquots of SUMOylated PML using SUMO protease 1 (Life Technologies) or LUNA in SUMO buffer provided by manufacturers. A SUMO-CHOP assay kit (Lifesensors) was used to identify isopeptidase activity.

### Indirect Immunofluorescence and Genome FISH

FLAG-LUNA and PML expression was detected with monoclonal mouse anti-FLAG M2 (1:500; Sigma-Aldrich) and goat anti-PML (1:500, clone N19; Santa Cruz Biotechnology) and detection with donkey anti-goat Alexa Fluor 488 nm and rabbit anti-mouse Alexa Fluor 594 nm antibodies (1:1,000; Millipore).

For Genome FISH experiments, infected CD34^+^ cells were infected and cultured on glass coverslips for 7 days prior to analysis using a previously published procedure (Tang and Maul, 2003). Briefly, cells were fixed in 4% paraformaldehyde and, following washing with PBS, were incubated with hybridization buffer (50% formaldehyde/10% dextran sulfate in PBS) for 1 hr at 37°C. Cells were then incubated with a fluorescently labeled HCMV cosmid DNA probe (Cy3-dCTP) for 90 s at 95°C in hybridization buffer followed by an overnight incubation at 37°C. Two washing steps in 2× saline sodium citrate buffer (5 min, 60°C) were followed by a PBS wash at room temperature and then staining as before to detect PML localization. Cells were visualized by confocal microscopy.

### Tissue Culture and Inhibitors

Primary CD34^+^ hematopoietic cells were isolated from apheresis blood packs harvested from cranulocyte-colony-stimulating factor-mobilized donors using CD34^+^ magnetic activated cell sorting (MACS) separation (Miltenyi Biotec). Alternatively, CD34^+^ cells isolated from granulocyte-colony-stimulating factor (G-CSF)-mobilized healthy donors were purchased for use in some experiments (Lonza).The inhibition of isopeptidase activity was achieved using G5 (1 μM unless stated otherwise; Calbiochem) by addition of the chemical directly to the culture media 8 hr post-transfection or 3 hr prior to the addition of LPS in reactivation studies.

### Latency Establishment and Co-culture Experiments

CD34^+^ hematopoietic cells were cultured for 4 hr in X-vivo 15 following isolation before HCMV infection (MOI = 5). After 3 hr, infected cells were washed and cultured in fresh X-vivo 15 media supplemented with 2.5 mM L-glutamine. After 7 days, media were exchanged for media containing cytokines (all from Peprotech) that promote Langerhans cell (LC) differentiation (TGF-β_1_ [0.5 ng/mL], Flt-3L and granulocyte-macrophage colony-stimulating factor [GM-CSF] [100 ng/mL], TNF-α [2.5 ng/mL], and SCF [20 ng/mL]). After 7 days, the formation of characteristic LC clusters occurred. To promote reactivation, immature LCs were stimulated with LPS (500 ng/mL; Sigma-Aldrich) to promote reactivation (Reeves et al., 2005a), which was detected at 24 hr post-infection by RT-PCR for IE gene expression or, alternatively, by assaying virus production from reactivating mature LCs following co-culture on a confluent monolayer of HFF with samples of supernatant taken at regular intervals and used to inoculate fresh HFF to test for infectious virus by indirect immuno-fluorescent staining. Viral titers were quantified by 50% tissue culture infective dose (TCID_50_).

### Analysis of SUMO Isoform Processing Activity of Recombinant LUNA

For analyses of SUMO processing activity, 6-histidine-labeled pre-SUMO-1 or biotin labeled poly-SUMO-2 chains (three to eight links; both from Boston Biochem) were incubated with either Senp2_CD_ (Supr1 catalytic domain; Boston Biochem) or rabbit reticulolysate TnT (Promega) reactions generated using a T3 primer on pCMVTag2B expression vectors used to express LUNA and LUNAg233c. FLAG-tagged proteins were immuno-precipitated and then eluted from the beads and then analyzed by silver stain for purity, and then equivalent levels of protein were incubated with 6-His-pre-SUMO-1 or biotin-poly-SUMO-2 (10% v/v), 24 μM dTT, 7 μl of TnT reaction, or 1 μl of SUMO protease-1 in a final volume of 20 μl (water) for 1 hr at 30°C. For studies on PML, PML was immuno-precipitated from cell lysates using goat anti-PML (clone N19, 1:250; Santa Cruz Biotechnology) and then incubated with E1 (SAE1/SAE2; 125 nM), E2 (UbcH9; 4 μM), and poly-SUMO-2 (5 μM) in SUMO reaction buffer (50 mM HEPES [pH 7.4], 5mM MgCl_2_ ATP, 0.6 mM DTT) for 1 hr at 30°C. For deSUMOylation experiments, SUMOylated PML beads were then split and incubated with either Senp2_CD_ or TnT reactions as described above. All reagents were from Boston Biochem unless otherwise stated.

### Nucleic Acid Isolation and Analysis

Total RNA was extracted from 10^6^ cells using the RNAeasy kit as described by the manufacturer (QIAGEN). Residual genomic DNA was removed by a DNase I digestion (Promega) followed by production of first-strand cDNA using the Promega RT system. Two methods were used to detect IE gene expression in samples. For the detection of endogenous HCMV IE RNA, cDNA derived from reactivating LCs was first amplified using 2× PCR MasterMix (Promega) containing DNA Polymerase, MgCl_2_ and dNTPs under the following conditions: 95°C (5 min), then 20–35 cycles of 94°C (1 min), 55°C (1 min), and 72°C (1 min), and then a final extension at 72°C for 10 min using IE72-specific sense primer 5′-CAT CCA CAT CTC CCG CTT AT-3′ and antisense primer 5′-CAC GAC GTT CCT GCA GAC TAT G-3′. Then 5 μl was added to a nested PCR reaction under the same conditions using 5′-GCG CCA GTG AAT TTC TCT TC and 5′-ACG AGA ACC CCG AGA AAG ATG. A 548 bp actin product was amplified using sense 5′-GCT CCG GCA TGT GCA-3′ and antisense 5′-AGG ATC TTC ATG AGG TAG T-3′ under the same PCR conditions except with the addition of MgCl_2_ (2.5 mM).

For studies of gene expression during experimental infection IE, UL138 and GAPDH gene expression was determined using a SYBR green qRT-PCR system (QIAGEN) with the primers for IE and UL138 as previously described (Petrucelli et al., 2009) and GAPDH (QIAGEN probe set). Reactions were set up using the QIAGEN SYBR green RT-PCR kit in accordance with the manufacturer’s protocol and the samples amplified at 50°C for 2 min, 95°C for 10 min, 60 cycles of 95°C for 15 s and 58°C for 30 s, melting curve analysis consisting of 95°C for 15 s and 60°C for 30 s, and a final step at 95°C for 15 s using the ABI 7500 Fast Real-Time PCR machine (Applied Biosystems).

### Western Blotting

Infected HFFs were harvested in Laemmli buffer and analyzed using SDS-PAGE. Protein expression was detected using anti-pp71 or anti-pp28 (1:1,000; Santa Cruz), anti-IE (1:1,000; Millipore), anti-GFP (1:1:1,000; Abcam), and anti-actin (1:1:1,000; Abcam).

### Analysis of Reactivation in THP1 PML KO Cells

THP1 cells with a PML KO have been described previously (Wagenknecht et al., 2015). The cells were used alongside control THP-1 cells in an HCMV-latency and reactivation model system (Lau et al., 2016). Viral DNA was quantified by qPCR (ABI Universal Mastermix) using gB primers (GAG GAC AAC GAA ATC CTG TTG GGC A and GRC GAC GGT GGA GAT ACT GCT GAG G) with the FAM-labeled probe (CAA TCA TGC GTT TCA AGA GGT AGT CCA quenched with BHQ1). GAPDH was used to determine relative levels of DNA in each sample, as previously published (Krishna et al., 2016).

### Statistical Analysis

A comparison of the mean was performed using Student’s t test, with p values < 0.05 regarded as indicating statistical significance.

## Supplemental Information

Document S1. Supplemental Experimental Procedures and Figures S1–S6

Document S2. Article plus Supplemental Information

## References

[bib1] Arcangeletti M.C., Vasile Simone R., Rodighiero I., De Conto F., Medici M.C., Maccari C., Chezzi C., Calderaro A. (2016). Human cytomegalovirus reactivation from latency: validation of a “switch” model in vitro. Virol. J..

[bib2] Bego M., Maciejewski J., Khaiboullina S., Pari G., St Jeor S. (2005). Characterization of an antisense transcript spanning the UL81-82 locus of human cytomegalovirus. J. Virol..

[bib3] Boutell C., Cuchet-Lourenço D., Vanni E., Orr A., Glass M., McFarlane S., Everett R.D. (2011). A viral ubiquitin ligase has substrate preferential SUMO targeted ubiquitin ligase activity that counteracts intrinsic antiviral defence. PLoS Pathog..

[bib4] Caswell R., Hagemeier C., Chiou C.J., Hayward G., Kouzarides T., Sinclair J. (1993). The human cytomegalovirus 86K immediate early (IE) 2 protein requires the basic region of the TATA-box binding protein (TBP) for binding, and interacts with TBP and transcription factor TFIIB via regions of IE2 required for transcriptional regulation. J. Gen. Virol..

[bib5] Cheng X., Kao H.Y. (2013). Post-translational modifications of PML: consequences and implications. Front. Oncol..

[bib6] Dollard S.C., Grosse S.D., Ross D.S. (2007). New estimates of the prevalence of neurological and sensory sequelae and mortality associated with congenital cytomegalovirus infection. Rev. Med. Virol..

[bib7] Dupont L., Reeves M.B. (2016). Cytomegalovirus latency and reactivation: recent insights into an age old problem. Rev. Med. Virol..

[bib8] Everett R.D., Chelbi-Alix M.K. (2007). PML and PML nuclear bodies: implications in antiviral defence. Biochimie.

[bib9] Everett R.D., Freemont P., Saitoh H., Dasso M., Orr A., Kathoria M., Parkinson J. (1998). The disruption of ND10 during herpes simplex virus infection correlates with the Vmw110- and proteasome-dependent loss of several PML isoforms. J. Virol..

[bib10] Everett R.D., Meredith M., Orr A. (1999). The ability of herpes simplex virus type 1 immediate-early protein Vmw110 to bind to a ubiquitin-specific protease contributes to its roles in the activation of gene expression and stimulation of virus replication. J. Virol..

[bib11] Geoffroy M.C., Chelbi-Alix M.K. (2011). Role of promyelocytic leukemia protein in host antiviral defense. J. Interferon Cytokine Res..

[bib12] Goodrum F., Reeves M., Sinclair J., High K., Shenk T. (2007). Human cytomegalovirus sequences expressed in latently infected individuals promote a latent infection in vitro. Blood.

[bib13] Groves I.J., Reeves M.B., Sinclair J.H. (2009). Lytic infection of permissive cells with human cytomegalovirus is regulated by an intrinsic ‘pre-immediate-early’ repression of viral gene expression mediated by histone post-translational modification. J. Gen. Virol..

[bib14] Hahn G., Jores R., Mocarski E.S. (1998). Cytomegalovirus remains latent in a common precursor of dendritic and myeloid cells. Proc. Natl. Acad. Sci. U S A.

[bib15] Hannoun Z., Greenhough S., Jaffray E., Hay R.T., Hay D.C. (2010). Post-translational modification by SUMO. Toxicology.

[bib16] Hargett D., Shenk T.E. (2010). Experimental human cytomegalovirus latency in CD14^+^ monocytes. Proc. Natl. Acad. Sci. U S A.

[bib17] Hickey C.M., Wilson N.R., Hochstrasser M. (2012). Function and regulation of SUMO proteases. Nat. Rev. Mol. Cell Biol..

[bib18] Humar A., St Louis P., Mazzulli T., McGeer A., Lipton J., Messner H., MacDonald K.S. (1999). Elevated serum cytokines are associated with cytomegalovirus infection and disease in bone marrow transplant recipients. J. Infect. Dis..

[bib19] Ishov A.M., Sotnikov A.G., Negorev D., Vladimirova O.V., Neff N., Kamitani T., Yeh E.T., Strauss J.F., Maul G.G. (1999). PML is critical for ND10 formation and recruits the PML-interacting protein daxx to this nuclear structure when modified by SUMO-1. J. Cell Biol..

[bib20] Kalejta R.F. (2008). Functions of human cytomegalovirus tegument proteins prior to immediate early gene expression. Curr. Top. Microbiol. Immunol..

[bib21] Kew V.G., Yuan J., Meier J., Reeves M.B. (2014). Mitogen and stress activated kinases act co-operatively with CREB during the induction of human cytomegalovirus immediate-early gene expression from latency. PLoS Pathog..

[bib22] Keyes L.R., Bego M.G., Soland M., St Jeor S. (2012). Cyclophilin A is required for efficient human cytomegalovirus DNA replication and reactivation. J. Gen. Virol..

[bib23] Keyes L.R., Hargett D., Soland M., Bego M.G., Rossetto C.C., Almeida-Porada G., St Jeor S. (2012). HCMV protein LUNA is required for viral reactivation from latently infected primary CD14^+^ cells. PLoS ONE.

[bib24] Kim K.I., Baek S.H., Jeon Y.J., Nishimori S., Suzuki T., Uchida S., Shimbara N., Saitoh H., Tanaka K., Chung C.H. (2000). A new SUMO-1-specific protease, SUSP1, that is highly expressed in reproductive organs. J. Biol. Chem..

[bib25] Krishna B.A., Lau B., Jackson S.E., Wills M.R., Sinclair J.H., Poole E. (2016). Transient activation of human cytomegalovirus lytic gene expression during latency allows cytotoxic T cell killing of latently infected cells. Sci. Rep..

[bib26] Lallemand-Breitenbach V., de Thé H. (2010). PML nuclear bodies. Cold Spring Harb. Perspect. Biol..

[bib27] Lau B., Poole E., Krishna B., Sellart I., Wills M.R., Murphy E., Sinclair J. (2016). The expression of human cytomegalovirus microRNA MiR-UL148D during latent infection in primary myeloid cells inhibits activin A-triggered secretion of IL-6. Sci. Rep..

[bib28] Lee H.R., Kim D.J., Lee J.M., Choi C.Y., Ahn B.Y., Hayward G.S., Ahn J.H. (2004). Ability of the human cytomegalovirus IE1 protein to modulate sumoylation of PML correlates with its functional activities in transcriptional regulation and infectivity in cultured fibroblast cells. J. Virol..

[bib29] Lee S.H., Albright E.R., Lee J.H., Jacobs D., Kalejta R.F. (2015). Cellular defense against latent colonization foiled by human cytomegalovirus UL138 protein. Sci. Adv..

[bib30] Legendre C., Pascual M. (2008). Improving outcomes for solid-organ transplant recipients at risk from cytomegalovirus infection: late-onset disease and indirect consequences. Clin. Infect. Dis..

[bib31] Liang Y.C., Lee C.C., Yao Y.L., Lai C.C., Schmitz M.L., Yang W.M. (2016). SUMO5, a novel poly-SUMO isoform, regulates PML nuclear bodies. Sci. Rep..

[bib32] Limaye A.P., Kirby K.A., Rubenfeld G.D., Leisenring W.M., Bulger E.M., Neff M.J., Gibran N.S., Huang M.L., Santo Hayes T.K., Corey L., Boeckh M. (2008). Cytomegalovirus reactivation in critically ill immunocompetent patients. JAMA.

[bib33] Lukashchuk V., McFarlane S., Everett R.D., Preston C.M. (2008). Human cytomegalovirus protein pp71 displaces the chromatin-associated factor ATRX from nuclear domain 10 at early stages of infection. J. Virol..

[bib34] Müller S., Hoege C., Pyrowolakis G., Jentsch S. (2001). SUMO, ubiquitin’s mysterious cousin. Nat. Rev. Mol. Cell Biol..

[bib35] Murphy J.C., Fischle W., Verdin E., Sinclair J.H. (2002). Control of cytomegalovirus lytic gene expression by histone acetylation. EMBO J..

[bib36] Owerbach D., McKay E.M., Yeh E.T., Gabbay K.H., Bohren K.M. (2005). A proline-90 residue unique to SUMO-4 prevents maturation and sumoylation. Biochem. Biophys. Res. Commun..

[bib37] Petrucelli A., Rak M., Grainger L., Goodrum F. (2009). Characterization of a novel Golgi apparatus-localized latency determinant encoded by human cytomegalovirus. J. Virol..

[bib38] Reeves M.B., Compton T. (2011). Inhibition of inflammatory interleukin-6 activity via extracellular signal-regulated kinase-mitogen-activated protein kinase signaling antagonizes human cytomegalovirus reactivation from dendritic cells. J. Virol..

[bib39] Reeves M.B., Sinclair J.H. (2010). Analysis of latent viral gene expression in natural and experimental latency models of human cytomegalovirus and its correlation with histone modifications at a latent promoter. J. Gen. Virol..

[bib40] Reeves M.B., Sinclair J.H. (2013). Circulating dendritic cells isolated from healthy seropositive donors are sites of human cytomegalovirus reactivation in vivo. J. Virol..

[bib41] Reeves M.B., Lehner P.J., Sissons J.G., Sinclair J.H. (2005). An in vitro model for the regulation of human cytomegalovirus latency and reactivation in dendritic cells by chromatin remodelling. J. Gen. Virol..

[bib42] Reeves M.B., MacAry P.A., Lehner P.J., Sissons J.G., Sinclair J.H. (2005). Latency, chromatin remodeling, and reactivation of human cytomegalovirus in the dendritic cells of healthy carriers. Proc. Natl. Acad. Sci. USA.

[bib43] Rossetto C.C., Tarrant-Elorza M., Pari G.S. (2013). Cis and trans acting factors involved in human cytomegalovirus experimental and natural latent infection of CD14 (+) monocytes and CD34 (+) cells. PLoS Pathog..

[bib44] Saffert R.T., Kalejta R.F. (2006). Inactivating a cellular intrinsic immune defense mediated by Daxx is the mechanism through which the human cytomegalovirus pp71 protein stimulates viral immediate-early gene expression. J. Virol..

[bib45] Saffert R.T., Penkert R.R., Kalejta R.F. (2010). Cellular and viral control over the initial events of human cytomegalovirus experimental latency in CD34+ cells. J. Virol..

[bib46] Scherer M., Reuter N., Wagenknecht N., Otto V., Sticht H., Stamminger T. (2013). Small ubiquitin-related modifier (SUMO) pathway-mediated enhancement of human cytomegalovirus replication correlates with a recruitment of SUMO-1/3 proteins to viral replication compartments. J. Gen. Virol..

[bib47] Shin E.J., Shin H.M., Nam E., Kim W.S., Kim J.H., Oh B.H., Yun Y. (2012). DeSUMOylating isopeptidase: a second class of SUMO protease. EMBO Rep..

[bib48] Sinclair J., Sissons P. (2006). Latency and reactivation of human cytomegalovirus. J. Gen. Virol..

[bib49] Söderberg-Nauclér C., Fish K.N., Nelson J.A. (1997). Reactivation of latent human cytomegalovirus by allogeneic stimulation of blood cells from healthy donors. Cell.

[bib50] Stanton R.J., Baluchova K., Dargan D.J., Cunningham C., Sheehy O., Seirafian S., McSharry B.P., Neale M.L., Davies J.A., Tomasec P. (2010). Reconstruction of the complete human cytomegalovirus genome in a BAC reveals RL13 to be a potent inhibitor of replication. J. Clin. Invest..

[bib51] Tang Q., Maul G.G. (2003). Mouse cytomegalovirus immediate-early protein 1 binds with host cell repressors to relieve suppressive effects on viral transcription and replication during lytic infection. J. Virol..

[bib52] Tavalai N., Stamminger T. (2011). Intrinsic cellular defense mechanisms targeting human cytomegalovirus. Virus Res..

[bib53] Tavalai N., Papior P., Rechter S., Leis M., Stamminger T. (2006). Evidence for a role of the cellular ND10 protein PML in mediating intrinsic immunity against human cytomegalovirus infections. J. Virol..

[bib54] Tavalai N., Adler M., Scherer M., Riedl Y., Stamminger T. (2011). Evidence for a dual antiviral role of the major nuclear domain 10 component Sp100 during the immediate-early and late phases of the human cytomegalovirus replication cycle. J. Virol..

[bib55] Wagenknecht N., Reuter N., Scherer M., Reichel A., Müller R., Stamminger T. (2015). Contribution of the major ND10 proteins PML, hDaxx and Sp100 to the regulation of human cytomegalovirus latency and lytic replication in the monocytic cell line THP-1. Viruses.

[bib56] Woodhall D.L., Groves I.J., Reeves M.B., Wilkinson G., Sinclair J.H. (2006). Human Daxx-mediated repression of human cytomegalovirus gene expression correlates with a repressive chromatin structure around the major immediate early promoter. J. Biol. Chem..

[bib57] Xu Z., Chau S.F., Lam K.H., Chan H.Y., Ng T.B., Au S.W. (2006). Crystal structure of the SENP1 mutant C603S-SUMO complex reveals the hydrolytic mechanism of SUMO-specific protease. Biochem. J..

[bib58] Zhuravskaya T., Maciejewski J.P., Netski D.M., Bruening E., Mackintosh F.R., St Jeor S. (1997). Spread of human cytomegalovirus (HCMV) after infection of human hematopoietic progenitor cells: model of HCMV latency. Blood.

