# Supplementary material for: A Virally Encoded DeSUMOylase Activity Is Required for Cytomegalovirus Reactivation from Latency
Source: Cell Rep. 2018 Jul 17;24(3):594–606. doi: 10.1016/j.celrep.2018.06.048 (PMC6077246; doi:10.1016/j.celrep.2018.06.048)
Supplement: Document S1. Supplemental Experimental Procedures and Figures S1–S6 [file mmc1.pdf]

Cell Reports, Volume *24*

Supplemental Information

A Virally Encoded DeSUMOylase Activity Is Required for Cytomegalovirus Reactivation from Latency

Emma L. Poole, Verity G. Kew, Jonathan C.H. Lau, Matthew J. Murray, Thomas Stamminger, John H. Sinclair, and Matthew B. Reeves

Supplemental Information

Supplemental Figures

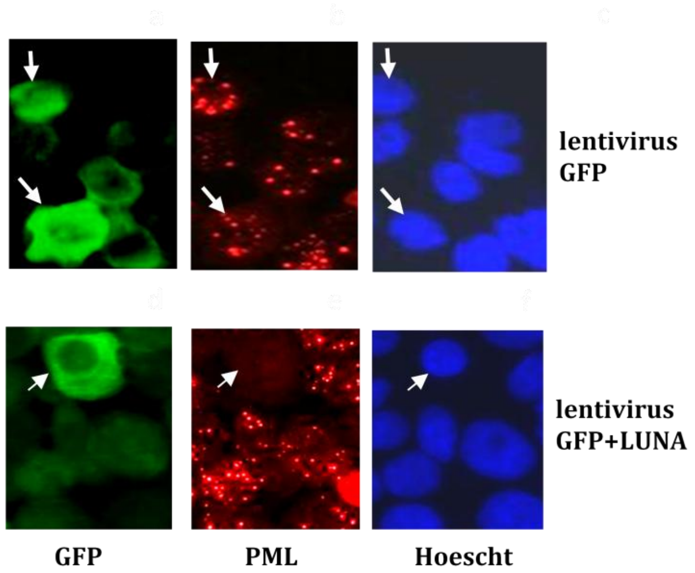

Figure S1. LUNA disrupts ND10 bodies in transduced myeloid cells. Related to Figure 2: U937 cells transduced with lentiviral vectors expressing GFP (a-c) or LUNA-IRES-GFP (d-f) were stained with anti-GFP (a,d), anti-PML (b,e) or Hoescht (c,f) and visualised by immunofluorescent microscopy.

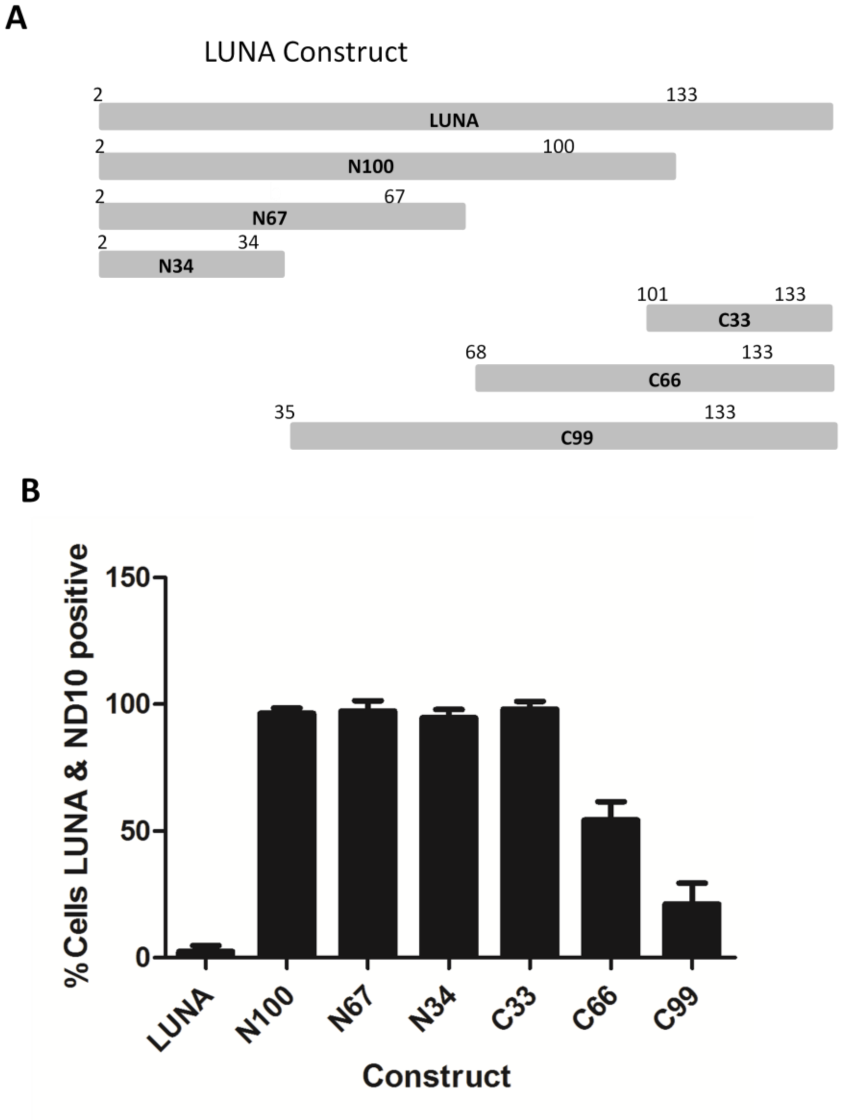

Figure S2. The activity of LUNA against ND10 bodies is dependent on the C terminus. Related to Figure 3
A-B) Summary of LUNA constructs transfected into fibroblasts scored for their ability to disperse ND10

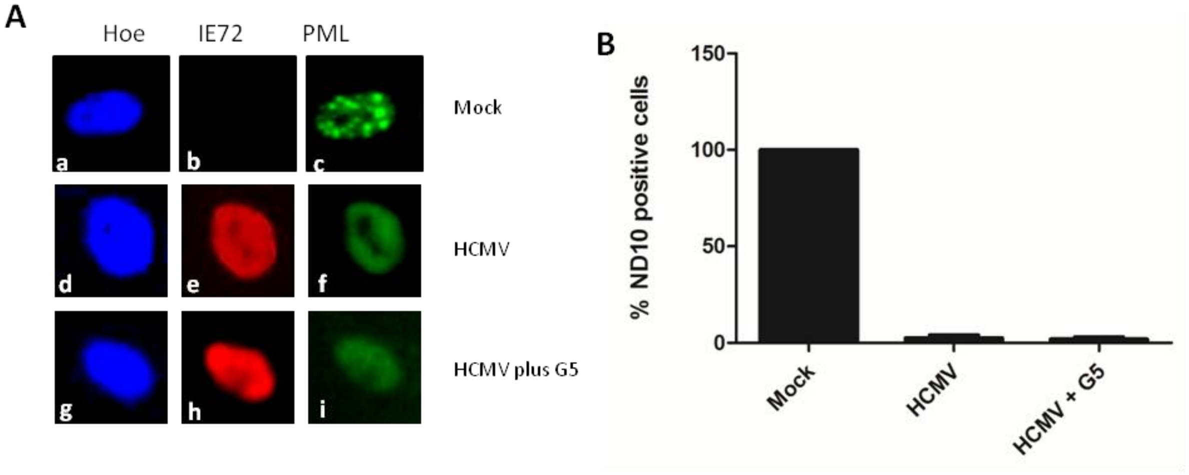

Figure S3. IE72 mediated disruption of ND10 bodies during lytic infection is not inhibited by isopeptidase inhibitors. Related to Figure 3: A-B) Mock (a-c) or HCMV infected HFF cells (d-i) were incubated with DMSO (d-f) or G5 isopeptidase inhibitor (g-i) and co-stained for IE (b,e,h) and PML (c,f,i) expression and counterstained with Hoescht (a,d,g) and the number of ND10 positive cells enumerated.

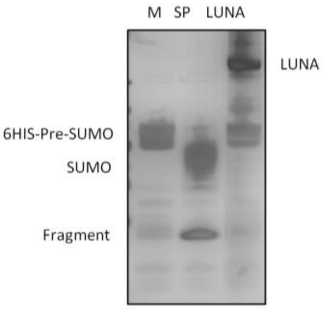

Figure S4. LUNA does not have detectable endopeptidase activity. Related to Figure 4: Pre-SUMO-1 was incubated with buffer (M), SENP2$_{CD}$ (SP), or LUNA (L) for 30 minutes and then analysed by silver staining for processing of pre-SUMO-1 to a mature form.

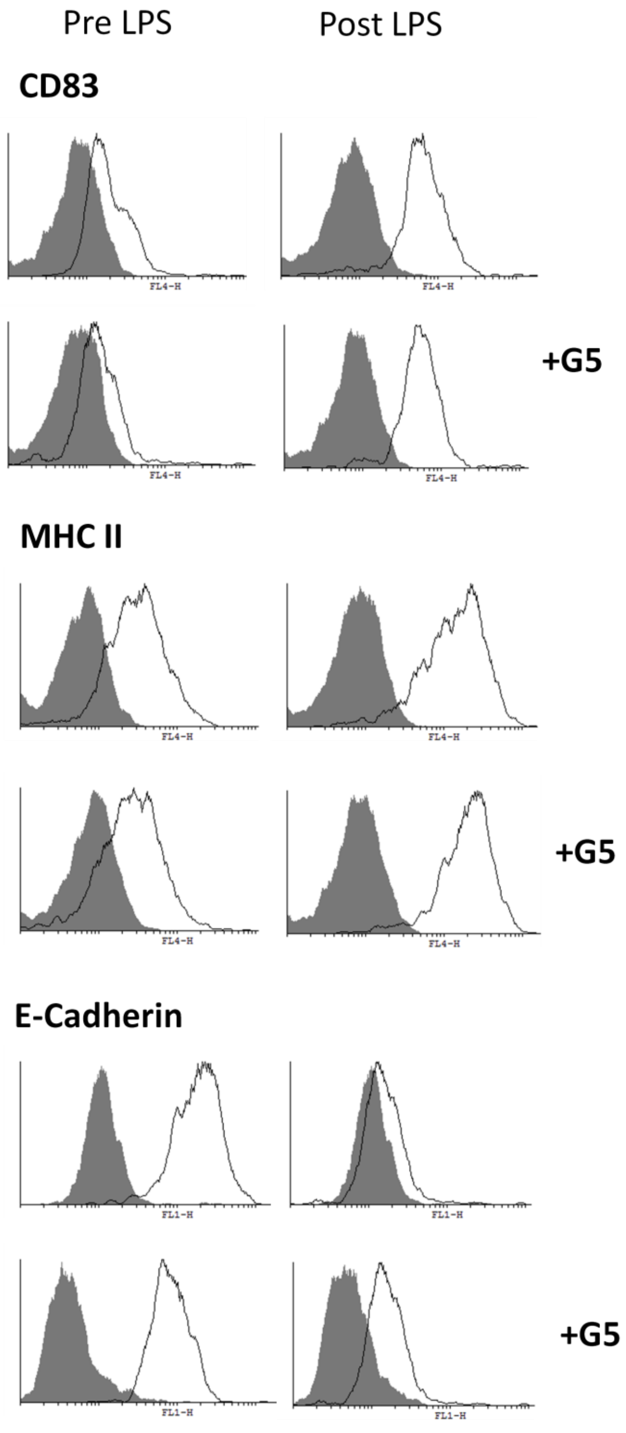

Figure S5. Isopeptidase inhibitor G5 does not prevent the differentiation of CD34+ cells to DCs. Related to Figure 6: Flow cytometric analysis of CD83, MHC class II and E-cadherin expression (open line) on CD34+ derived DCs pre and post LPS stimulation. Prior to LPS cells were treated with G5 (2.5uM) for 1 hour for comparison. Isotype controls shown (Filled histogram).

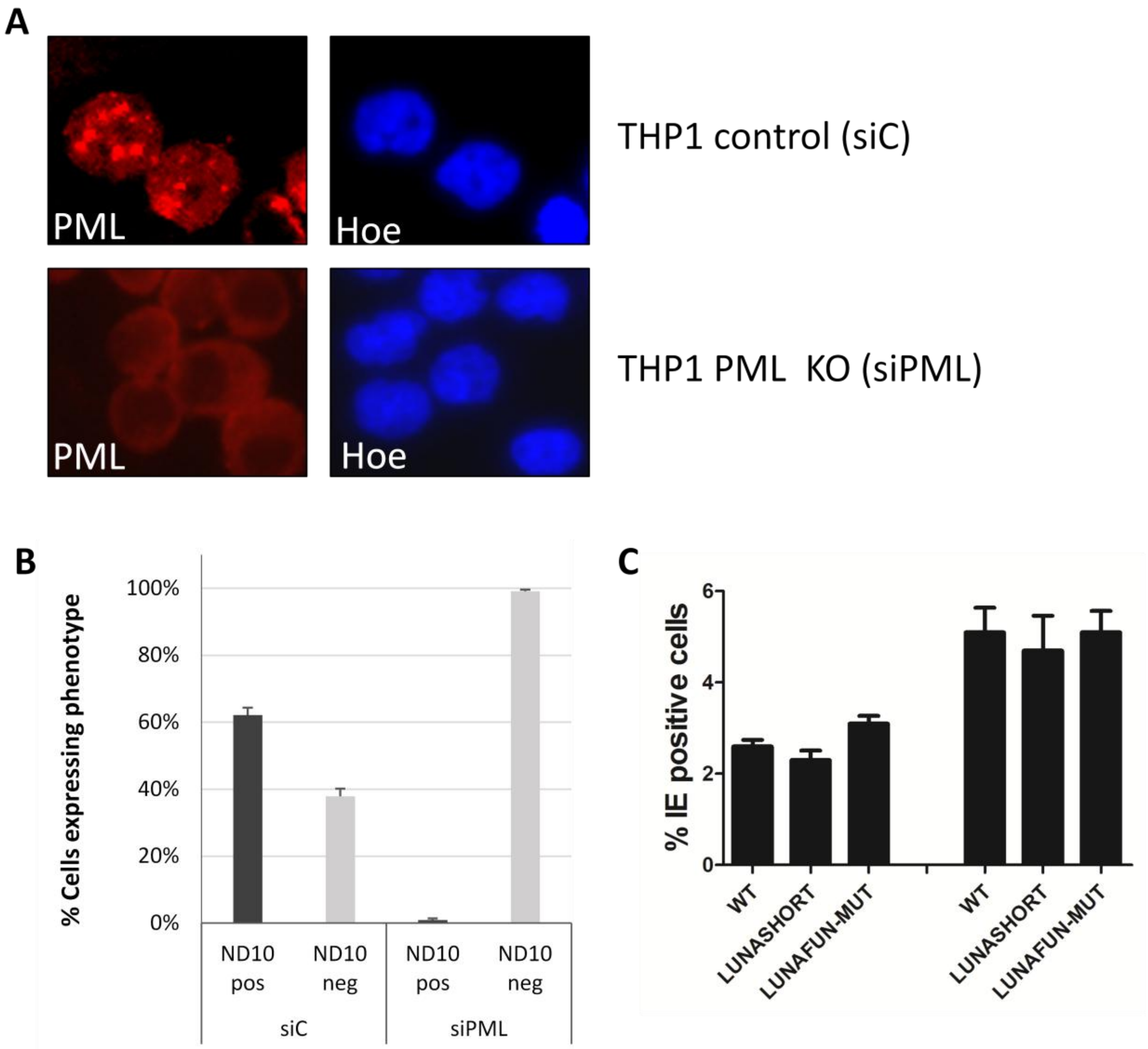

Figure S6. siPML treated THP1 cells have no PML/ND10 bodies and are equally to susceptible to infection with wild type and LUNA mutant viruses. Related to Figure 7 A-B) THP1 cells transduced with retroviral constructs expressing control (siC) or PML (siPML) targeting siRNAs were stained for PML (A) and the number of PML/ND10 bodies was enumerated (B). **C)** THP1 cells expressing siC or siPML were differentiated with PMA for 72 hours and then infected (MOI=0.1) with Merlin (WT), LUNA protein deletion ($LUNA_{SHORT}$) or LUNA catalytic dead mutant ($LUNA_{FUN\text{-}MUT}$) and immuno-stained for IE expression 24 hours post infection. Infected cells were quantified and expressed as a percentage of total number of nuclei.

Supplemental Experimental Procedures

Plasmids: The LUNA truncations were generated using PCR primers to amplify fragments of LUNA DNA and were ligated into the pCMVTag2B vector using BamHI/HindIII restriction sites as described for full length LUNA which were introduced by PCR. All plasmids were sequence verified.

Indirect IF: Infected cells were rinsed in PBS and fixed for 10 minutes in 4% paraformaldehyde at room temperature. After permeabilizing with 0.1% Triton-X-100 in PBS, cells were incubated with monoclonal mouse anti-IE antibody (Millipore, Billerica, MA) (1:1000 dilution in PBS) for 1 hour at room temperature. After washing with PBS, the bound antibodies were detected using Alexafluor 594nm (Millipore, Billerica, MA)-conjugated goat anti-mouse immunoglobulins (1:1000 dilution in PBS) together with nuclear stain Hoechst (1:1000 dilution in PBS) in the dark for 1 hour at room temperature. After washing with PBS, infected cells were visualised using a Nikon immunofluorescence microscope and were quantified using ImagePro WCIF ImageJ software (National Institutes of Health). Percentage infection was calculated by dividing the number of infected cells (red) by the total number of cells (blue) from at least 4 fields of view.

Tissue Culture: Human foreskin fibroblasts (HFFs) were maintained in Eagle's minimal essential medium containing 10% fetal calf serum (EMEM-10) (Sigma-Aldrich, Poole, UK) and incubated at 37°C and in 5% CO_2following standard procedure for tissue culture and propagation of HCMV stocks.

Cell surface phenotype flow cytometry analysis: 10^5 cells were pelleted at 400g for 5 minutes and were then re-suspended in the residual volume. Then cells were incubated for 20 minutes with 3ul of marker specific antibodies or the appropriate isotype control: fluorescein isothiocyanate (FITC)-conjugated mouse anti-human CD207 or CD1a antibodies, APC-conjugated mouse anti-human CD83 or anti HLA-DR antibodies or an Alexafluor 488nm conjugated E-Cadherin antibody (R&D systems). Following washing in 10× volumes of PBS, the cells were pelleted at 400g for 5 minutes and were re-suspended in 500μl of phosphate buffered saline (PBS) before analyzing by flow cytometry (BD FACScalibur or BD FACSsort). Data handling was performed using Cyflogic software. All Antibodies were from BD Life Sciences (Frankin Lakes, NJ) unless otherwise stated.
